# Mango Fruit Load Estimation Using a Video Based MangoYOLO—Kalman Filter—Hungarian Algorithm Method

**DOI:** 10.3390/s19122742

**Published:** 2019-06-18

**Authors:** Zhenglin Wang, Kerry Walsh, Anand Koirala

**Affiliations:** 1 Centre for Intelligent Systems, Central Queensland University, Rockhampton North 4701, Australia; 2 Institute of Future Farming Systems, Central Queensland University, Rockhampton North 4701, Australia; a.koirala@cqu.edu.au

**Keywords:** crop load, Kalman filter, deep learning, YOLO, Hungarian assignment, tree fruit load

## Abstract

Pre-harvest fruit yield estimation is useful to guide harvesting and marketing resourcing, but machine vision estimates based on a single view from each side of the tree (“dual-view”) underestimates the fruit yield as fruit can be hidden from view. A method is proposed involving deep learning, Kalman filter, and Hungarian algorithm for on-tree mango fruit detection, tracking, and counting from 10 frame-per-second videos captured of trees from a platform moving along the inter row at 5 km/h. The deep learning based mango fruit detection algorithm, MangoYOLO, was used to detect fruit in each frame. The Hungarian algorithm was used to correlate fruit between neighbouring frames, with the improvement of enabling multiple-to-one assignment. The Kalman filter was used to predict the position of fruit in following frames, to avoid multiple counts of a single fruit that is obscured or otherwise not detected with a frame series. A “borrow” concept was added to the Kalman filter to predict fruit position when its precise prediction model was absent, by borrowing the horizontal and vertical speed from neighbouring fruit. By comparison with human count for a video with 110 frames and 192 (human count) fruit, the method produced 9.9% double counts and 7.3% missing count errors, resulting in around 2.6% over count. In another test, a video (of 1162 frames, with 42 images centred on the tree trunk) was acquired of both sides of a row of 21 trees, for which the harvest fruit count was 3286 (i.e., average of 156 fruit/tree). The trees had thick canopies, such that the proportion of fruit hidden from view from any given perspective was high. The proposed method recorded 2050 fruit (62% of harvest) with a bias corrected Root Mean Square Error (RMSE) = 18.0 fruit/tree while the dual-view image method (also using MangoYOLO) recorded 1322 fruit (40%) with a bias corrected RMSE = 21.7 fruit/tree. The video tracking system is recommended over the dual-view imaging system for mango orchard fruit count.

## 1. Introduction

A mango harvest yield prediction is ideally made six weeks before harvest, after the period of fruit drop in fruit development, to inform harvesting and marketing decisions [1]. However, mango production varies between seasons due to a biennial bearing habit [2] and to environmental (e.g., temperatures) and management (e.g., pruning) variables, and thus the relationship between historical production and the current season can be poor. Further, mango tree fruit load is variable within a given orchard and season, such that a statistically valid estimate of pre-harvest crop load requires the assessment of a large number of trees [3]. 

Given the required number of trees for a statistically valid estimate of average fruit load, the use of field counts by human operators is impractical [3]. The use of machine vision (MV) based on RGB images of tree canopies has been trialed for tree fruit yield estimation by a number of research groups. When fruit colour is distinct to foliage, a simple segmentation method using colour features can be successful for fruit detection. For example, Zaman et al. [4] used colour features in the estimation of blueberry yield, with a high R^2^ of 0.99 achieved, likely due to the distinct blue colour of the fruit. Zhou et al. [5] used red colour features in the detection of mature ‘Gala’ apples, with a reported R^2^ of 0.8. However, in other applications, fruit may be green or have varied colours. Also, perceived object colour is influenced by ambient light. Annamalai and Suk Lee [6] achieved a R^2^ of only 0.76 on human count of images using a MV technique based on colour to count mature citrus fruit in images of tree canopies.

A number of researchers have given attention to the shape and texture features in the detection of fruit. Low level feature detection algorithms exploit features such as object edges [7], corners [8], blobs [9], and ridges [10]. Various high-level feature extraction algorithms have also been used to evaluate small regions of interest, generating a set of feature descriptors (or feature vectors). Popular algorithms include Scale Invariant Feature Transform (SIFT) [11], Speeded Up Robust Features (SURF) [12], Histogram of Oriented Gradients (HOG) [13], and Local Binary Patterns (LBP) [14]. For example, Kurtulmus et al. [15] extracted circular Gabor texture features for green citrus yield estimation. Zhou et al. [5] used the shape features in the estimation of grape yield. Linker et al. [16] and Qureshi et al. [17] utilised arc features to estimate the number of green apples and mango fruit, respectively, in an orchard. In these reports, colour features can be a supporting, but not dominant, criterion.

Deep learning neural networks have an object-recognition ability that can outperform humans, as demonstrated in [18,19,20,21]. The models are essentially black box in that it is unclear what image features are used. Deep learning algorithms have been recommended for tree fruit detection, as reviewed by Koirala, et al. [22]. The first report was made by Sa, et al. [23] using the Faster Region- Convolutional Neural Networks (Faster-RCNN) algorithm [24] to detected multi-coloured (green, red, or yellow) capsicum fruit. Chen et al. [25] used a Fully Convolutional Network [26] to count fruit in apple and orange orchards. Bargoti and Underwood [27] used Faster-RCNN and transfer learning to estimate the yield of apple, mango, and almond orchards. 

The use of MV in the estimation of mature mango fruit yield was first reported by Payne, et al. [28], based on a segmentation approach that relied on colour features. A R^2^ of 0.74 and bias corrected RMSE of 7.7 fruit per tree was achieved. The authors reported on the impact of varying ambient light on detection accuracy, and night imaging with artificial lighting was adopted in their subsequent research [29]. They also reported the use of SIFT features to improve detection accuracy (R^2^ improved from 0.78 to 0.85) [17]. The problems of imaging in daylight were addressed using Xe flash lamps and very short exposure times [30]. These authors employed Faster-RCNN and ‘multi-view’ imagery to obtain a R^2^ > 0.9 on mango fruit count per tree. In a continuation study, Bargoti and Underwood [27] reported a F1-score of 0.908 and precision of 0.958 achieved for the estimation of mango yield per tree. Anderson et al. [3] compared the estimates for several orchards, and concluded that the method based on multi-view imaging and Faster-RCNN gave a better result than a method based on satellite imagery [31] or methods based on manual counts of a sample of trees. Subsequently, Koirala et al. [32] reported improved results for mango fruit load estimation using a You Only Look Once (YOLO) based deep learning architecture [33,34] termed ‘MangoYOLO’, relative to the Faster R-CNN architecture (F1 score of 0.968 and 0.945 for MangoYOLO and Faster-RCNN, respectively, on the same image set). In the current study, we adopt MangoYOLO as the state-of-art detection and localization algorithm for mango fruit.

A number of the previous MV based imaging systems for orchard assessment have employed a dual-view imaging approach in which one image from each side of each tree, centered on the tree trunk, is acquired (e.g., [35]). However, dual-view imaging gives only one perspective of each side of the tree, with a proportion of fruit hidden from view. The proportion of ‘hidden’ fruit varies with canopy density, and thus Koirala et al. [32] relied on the use of a correction factor based on a human count of fruit on a sample of trees for each orchard. The highest factor reported by Koirala, et al. [32] for a given mango orchard was 2.43, associated with large trees and dense foliage, and the lowest factor was 1.05, associated with open canopies and sparse foliage. However, ‘dual view’ imaging can result in a high number of unobserved fruit per tree (i.e., occluded fruit), and the ultimate estimate of orchard fruit count is sensitive to the estimate of the correction factor.

Imaging of each tree from multiple perspectives, for example from a camera on a vehicle moving down the orchard row, allows for detection of a greater proportion of fruit on a tree. In this case there is less sensitivity to error in the estimate of the correction factor, but a system is required to track fruit between images to avoid multiple counting of fruit. Liu et al. [36] summarized three major issues causing over counting in video based fruit yield estimation—(1) double count of the same fruit detected in consecutive frames; (2) double count of a fruit which is tracked across several frames, not detected in a frame due to occlusion or temporary failure of detection, and then detected and tracked again; and (3) double count of fruit seen from both sides of a tree. Another category can be added—(4) mis-counting of new fruit appearing close to the position of a fruit that was present in the previous frame but absent in the current frame, with mis-assignment of the new fruit to that fruit.

Several methods have been applied to address the above issues. The first approach is to register fruit based on a 3D point cloud. The 3D point cloud can be developed using combinations of Light Detection and Ranging (LiDAR) sensors, high precision Global Navigation Satellite System (GNSS), stereo imaging, and Structure from Motion (SfM). Other methods such as Kernelized Correlation Filters (KCF) extract features of the target object in the initial frame and then seek the best approximation in the sequent frames via linear or nonlinear regression, so as to track the object [37]. This method is effective in tracking a single distinctive object in a scene. In the proposed application of tree fruit load estimation, each image contains many objects (fruit) with similar features, such that the method is unlikely to work well. A more common approach is to use a state-of-the-art object detection method such as Faster-RCNN or YOLO to detect multiple fruit, and then apply a tracking algorithm such as optical flow [38] or Kalman filter [39]. A correlation may be needed to correlate the same fruit in successive frames, as provided by the Hungarian algorithm. 

A number of studies have attempted to acquire 3D information on fruit position. Using a high-performance GNSS to improve the 3D reconstruction accuracy, Moonrinta et al. [40] utilised SfM to generate a point cloud for pineapple orchards to track and count pineapple fruit. Wang et al. [41] employed stereo imaging and high-precision GNSS geolocation to generate a 3D structure of apple canopies and estimate apple yield, with a registration threshold of 5 cm for fruit imaged in sequential views on the same side of the tree, and 16 cm for fruit imaged from the opposite side of the row. The platform travelled at a very low speed of 0.9 km/h. 

Stein et al. [30] and Bargoti and Underwood [27] used a platform travelling at approximately 5 km/h to acquire images at 5 fps (‘multi-view’ imaging) and employed the Hungarian assignment to track fruit. A GNSS-inertial navigation system (GNSS/INS) was used to provide the absolute and relative camera poses for each image, and then the change of camera position (termed ‘epipolar’) was used to describe the translation of the whole image. The 3D localisation of each fruit achieved using eipipolar projection was then projected onto a LiDAR tree mask to provide assignment of fruit to individual trees. 

Gan et al. [42] utilised a system consisting of GNSS, inertial measurement unit (IMU), wheel encoders, and LiDAR to achieve Simultaneous Localization And Mapping (SLAM), with an extended Kalman filter to improve the reliability and accuracy of localization. The system tracks fruit in a real-world coordinate system. Halstead et al. [43] used a simple Intersection over Union (IoU) technique to correlate (track) fruit in frame sequence for capsicum fruit quantity estimation, with the prerequisite that the video was acquired at a high speed (30 fps) and the platform (robot arm) moved at a low speed, such that the fruit had little movement between successive frames. 

Use of a 3D localisation system in which each fruit is assigned a universal world coordinate position can ease the tracking issue. However, issues remain—(i) each sensor will carry a measurement error and the accumulating errors can be significant; (ii) fusion of measurements from different sensors can be an issue, such as the registration of LiDAR depth to RGB images, or the use of IMU information to enhance GPS accuracy and camera position; (iii) the optical flow method assumes that the pixel intensity of the target object remains similar in the frame sequence, and the neighbouring pixels have similar motion, such that the optical flow method is vulnerable to large motions, occlusion, illumination changes, and changes of the appearance of the objects; and (iv) SfM employs various feature extraction methods to reconstruct a 3D point cloud, but difficulties occur in choice of the best match features, the exclusion of outliers, and there may be an inadequate number of feature points if the movement between two frames is large; and (v) the combined set of high precision sensors results in an expensive system.

A lower cost, simplified system for tracking objects across image frames must, however, cope with camera rotation. For the orchard application, the moving platform will introduce some rotation to the camera. Stein et al. [30] described this issue as platform oscillation, and assumed that the translation of the camera could be applied to individual fruit in the image. However, camera rotation will impact the position of fruit across the image differently, with increasing effect further from the centre of the image. We propose that a Kalman filter can be used to predict fruit movement, accommodating camera rotation, and change in scale (due to camera-to-fruit distance changing), as well as the linear translation of position. The rotation and scale can be treated as a process noise, with the Kalman filter used to obtain optimal predictions of individual fruits in future frames based on their trajectories in previous frames. In such a method, fruit at different locations within the image are assigned different horizontal and vertical speeds, rather than a universal global translation. If a stable Kalman filter model is absent for a given fruit (e.g., if it was not detected in previous frames), the fruit can be assigned a model from the neighbouring fruit. The prediction error from the Kalman filter can be then mitigated by a Hungarian assignment algorithm.

In summary, the estimation of fruit load using the dual view approach suffers from sensitivity to the estimation of a correction factor for hidden fruit, related to the magnitude of this factor. Use of a tracking count using video will decrease the proportion of unseen fruit, and can remove the need for accurate spatial location of every tree as required for dual view imaging. In the current study we couple MangoYOLO, as the state-of-the-art detection and localisation algorithm, to the use of the Hungarian algorithm with a Kalman filter to track and count mango fruit in a video of mango trees. The novelty of this approach lies in context of a lower complexity and cost solution contrast to the epipolar projection procedure proposed by Bargoti and Underwood [27] or the high-precision GNSS based registration proposed by Wang, et al. [41]. 

## 2. Materials and Methods

### 2.1. Imaging

Mango orchards of cultivar Calypso^tm^ (latitude -25.14, longitude 152.37) and Honey Gold^tm^ (latitude -23.02, longitude 150.63) were imaged. These are orchards GE1 and HG B, respectively, in the study of [44] and [32] and are described in those papers in terms of tree density and size. 

**Figure 1:**
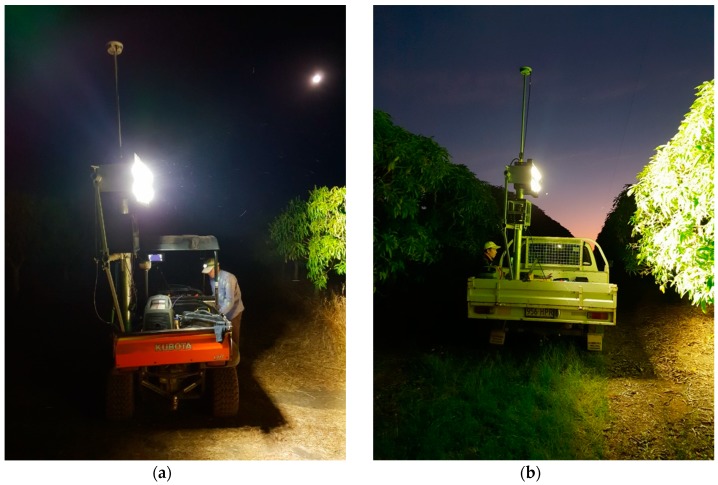
Automated imaging platform, involving a GNSS device (on pole), a 720 W LED floodlight, and a camera, powered by an inverter-generator and mounted on a farm vehicle ((**a**) buggy or (**b**) utility), operated at 5 km/h.

The imaging platform was previously reported in [32,35] and evolved from the systems used by Payne, et al. [29]. Briefly, the automated imaging platform (Figure 1) consisted of a frame mounted to a farm vehicle carrying a 720 W LED flood light and a Basler acA2440-75um machine vision camera (maximum 75 fps, operated at 10 fps, 5 Mp, 2448 × 2048 pixels) with a Goyo (GM10HR30518MCN) lens (5 mm focal length). The camera was set at a F/# of 1.8 with an exposure of 2.5 ms for the capture of both images and video. The distance from the camera to canopy was typically 2 m. As a one-off task, every tree was geo-located to an accuracy of within 2 cm using a Leica GS-14 unit operating on a Continuously Operating Reference Stations (CORS) network. For tree imaging, the camera was triggered with reference to the pre-acquired GNSS position of trees. The images and video are available as a supplementary data file to this manuscript. The imaging rig operated after sunset at a speed of about 5 km/hr, such that there were around 30 frames per tree and a shift of approximately 20 pixels per frame. The orchard land was relatively flat, so there was minor vertical movement of the vehicle, and the vertical speed was assumed to be zero. 

Images and video frames (10 fps) were scaled to 1024 × 1024 pixels for the use in MangoYOLO. The MangoYOLO architecture and model weights were adopted from Koirala, et al. [32]. The confidence threshold was set at 0.24 and the NMS threshold at 0.45, based on the previous work of Koirala, et al. [32]. 

The MangoYOLO deep neural network was trained on a CQUniversity High Performance Computing (HPC) facility graphics node with the following specifications—Intel^®^ Xeon^®^ Gold 6126 (12 cores, 2600 MHz base clock) CPU, NVIDIA^®^ Tesla^®^ P100 (16 GB Memory, 1328 MHz base clock, 3584 CUDA cores) GPU, Red Hat Enterprise Linux Server 7.4 (Maipo) and 384 GB RAM, CUDA v9.0, cuDNN v7.1.1, OpenCV v3.4.0, Python v2.7.14, and GCC v4.8.5.

The MangoYOLO detector was run on a common laptop (Acer Aspire VN7-593G, New Taipai, Taiwan), with the specification of—Intel^®^ Core™ i7-7700HQ CPU @ 2.80 GHz, 32 GB RAM, NVIDIA GeForce GTX 1060 GPU (1506 MHz GPU clock) with 6 GB dedicated memory, 64 bit Windows 10 Pro, CUDA v10.0, cuDNN v7.0.5, and OpenCV v4.0. The algorithm was implemented based on the OpenCV library and programmed in C/C++ language. The processing speed was 3.7 s/frame (407 s for 110 frames).

### 2.2. Fruit Counting

#### 2.2.1. Dual-View Fruit Counting

**Figure 2:**
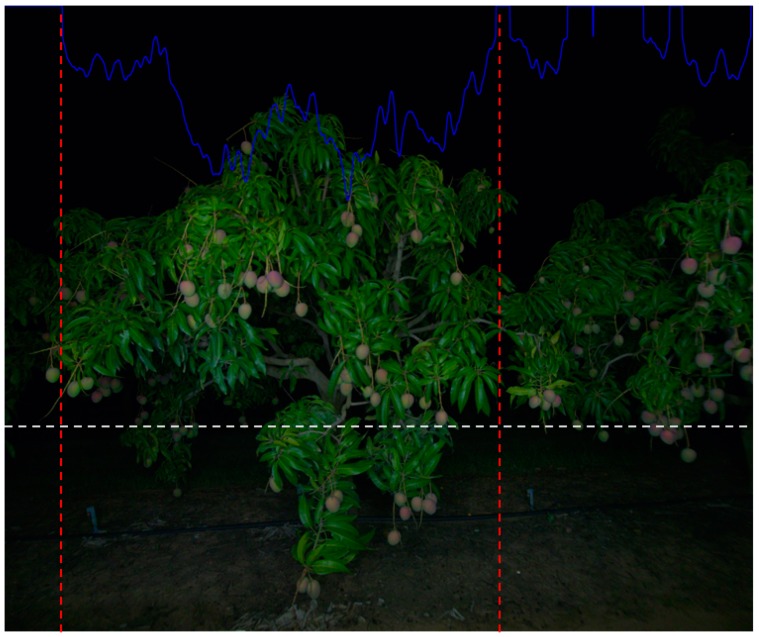
Auto tree segmentation. The Otsu’s method was applied to the image above the white line. The sum of intensity of each pixel column x -1 is illustrated as the blue line (zero at image top). Moving away from the image center in each direction, two near zero minima were identified (illustrated with red lines) and used to segment each tree. Canopy segmentation was based on the top two thirds of the image (as denoted by the white horizontal dash line, approximately 1.5 m above ground), as this part of the image carried the sky silhouette of the canopy and the max width of the canopy.

For ‘dual-view’ estimates, the method of Koirala, et al. [32] was adopted. Briefly, two dual-view images (one per tree side) were acquired for each tree. An auto segmentation method was used to delimit trees by exploiting their contours. The Otsu’s method [45] was applied to the grayscale images to remove pixels (set the pixel values to zeros) below the optimum threshold. Then, a sum of the intensity of each column was projected onto the horizontal dimension (see blue line in Figure 2). The points close to zero were considered as the delimiters of two neighboring trees. Thus, trees were segmented if they were separated by a gap or only slightly touched each other; otherwise, images were cropped with a fixed margin. The MangoYOLO model developed by Koirala, et al. [32] with a reported F1 score of 0.968 and Average Precision of 0.983, was then used to detect the fruit in the segmented images, per tree. Finally, individual tree counts were summed to obtain a final yield for the tree row.

#### 2.2.2. Video Based Fruit Tracking and Counting

MangoYOLO and Kalman Filter.

The MangoYOLO approach was again used to detect fruit location, with the centre point of the output bounding box (x, y) used as the fruit location. The Kalman filter was used to track and predict fruit position for fruit which were not present in a subsequent frame. The variation of vehicle speed and the minor scaling and rotation variations of the image were considered as process noise. A simple model was established involving four measurements—fruit location (x, y) and travel horizontal and vertical speeds ($v_{x}$ and $v_{y}$). The transition state matrix F and the measurement matrix H are:

(1) $$F=\left[\begin{array}{cccc} 1 & 0 & dt & 0\\ 0 & 1 & 0 & dt\\ 0 & 0 & 1 & 0\\ 0 & 0 & 0 & 1 \end{array}\right]$$

(2) $$H=\left[\begin{array}{cccc} 1 & 0 & 0 & 0\\ 0 & 1 & 0 & 0 \end{array}\right]$$

where dt is the time (frame) interval, which was set at 1. The choices of the process and measurement noise covariances are empirical. A small covariance of the process noise leads to accurate estimation, but requires more updates, resulting in a long lag time to build a precise prediction model. To provide for a rapid response, a relatively large covariance of 1.0 was chosen for the process noise. The measurements of fruit locations are fairly precise, and thus a relatively small covariance **R** of 0.1 was set for the measurement noise. The state vector is $\left(x,\;y,v_{x},v_{y}\right)$, denoted by $x_{k}$.

The Kalman filter has two major steps—predict and update. At the prediction stage, a priori state prediction is modelled by:

(3) $$x_{k|k-1}=Fx_{k-1|k-1}$$

where $x_{k-1|k-1}$ is previous location of a fruit. $x_{k|k-1}$ is the intermediate predicted location without considering the Kalman gain. Meanwhile, a priori predicted error covariance can be calculated as:

(4) $$P_{k|k-1}=FP_{k-1|k-1}F^{T}+Q$$

where Q is the covariance of the process noise. The Kalman gain can be calculated as:

(5) $$K_{k}=P_{k|k-1}H^{T}{\left(HP_{k|k-1}H^{T}+R\right)}^{-1}$$

If the fruit is detected in the current frame, an update process is needed. Given the fruit new measured location is $z_{k}$, the measurement residual $r_{k}$ is:

(6) $$r_{k}=z_{k}-Hx_{k|k-1}$$

Then a posteriori state estimate is updated as:

(7) $$x_{k|k}=x_{k|k-1}+K_{k}r_{k}$$

and the posteriori error covariance is

(8) $$P_{k|k}=\left(I-K_{k}H\right)P_{k|k-1}$$

where I is an Identity matrix.

However, even if a large covariance of process noise is chosen to promote the fast convergence of modelling, the Kalman filter still requires at least four continuous updates (four measurements) to obtain a relatively accurate location prediction model. In practice, some fruit only appear a few (< 4) times in the whole video, and thus the above standard Kalman filter is not applicable to those fruit. In such a case, a speed $\left(\begin{array}{c} v_{x}\\ v_{y} \end{array}\right)$ can be borrowed from a neighbouring fruit (where that fruit has been updated four or more times and a stable location prediction model has been established). Using the borrowed $\left(\begin{array}{c} v_{x}\\ v_{y} \end{array}\right)$, an artificial measurement can be provided as:

(9) $$z_{k}=Hx_{k-1|k-1}+\left(\begin{array}{c} v_{x}\\ v_{y} \end{array}\right)$$

If no “stable” fruit can be found, as occurs with the first four frames, a global speed $\left(\begin{array}{c} v_{x}=20\\ v_{y}=0 \end{array}\right)$ was used, based on the assumption that the platform travels at a speed of 5 km/h on flat ground. 

A process was required to avoid repeat counts for the situation where a fruit was not detected in one or more frames, due to occlusion by leaves or branches or a false negative, but then reappears. Once a stable location prediction model is built, the location of a given fruit can be anticipated in subsequent frames. A fruit reappearing in the expected position of a fruit within 15 frames of its last detection was assumed to be that fruit.

Hungarian Filter.

It is a challenge to match individual fruit between neighbouring frames, when there are many fruit in each video frame. The classic Hungarian method is a combinatorial optimisation algorithm that solves this assignment problem. The Euclidean distance of a fruit in two successive frames is used for the registration of a fruit if it occurs in both. The cost matrix can be then constructed with the element equaling:

(10) $$C_{i,j}=\sqrt{{\left(x_{i}-x_{j}\right)}^{2}+{\left(y_{i}-y_{j}\right)}^{2}}$$

The Hungarian algorithm aims to find a minimum total cost of assignment, which can be modelled as:

(11) $$\begin{array}{c} \min\overset{m}{\underset{i=1}{\sum }}\overset{n}{\underset{j=1}{\sum }}C_{i,j}x_{i,j}\\ s.t.\;\overset{m}{\underset{i=1}{\sum }}x_{i,j}=1,\;\overset{n}{\underset{j=1}{\sum }}x_{i,j}=1\;\;and\;x_{i,j}\in \left\{0,\;1\right\} \end{array}$$

where m and n are the numbers of tracked and new fruit. 

For the fruit count application, the algorithm was used to calculate the minimum total cost to correlate fruit between two subsequent frames. Each job (new fruit) was assigned to one worker (tracked fruit) and each worker was assigned one job (known as ‘bijection correspondence’). The number of new fruits can be either more or less than the number of (continuing) tracked fruit, although in most case it should be less. Given a travel speed of the imaging platform of 5 km/h, there is a horizontal shift of 20 pixels per frame. Therefore, a maximum distance threshold (redundancy considered) was applied to each assignment to judge if it is a valid assignment. If a fruit in the first frame was associated to a fruit in the second frame, the fruit was considered tracked, and its location in Frame 2 was used to update the Kalman filter. Otherwise, the predicted location from the Kalman filter was used as its new location and an invisible counter related to the fruit was increased by one. Unassigned fruit in Frame 2 were identified as new fruit and a new Kalman filter was created and appointed to the fruit.

**Figure 3:**
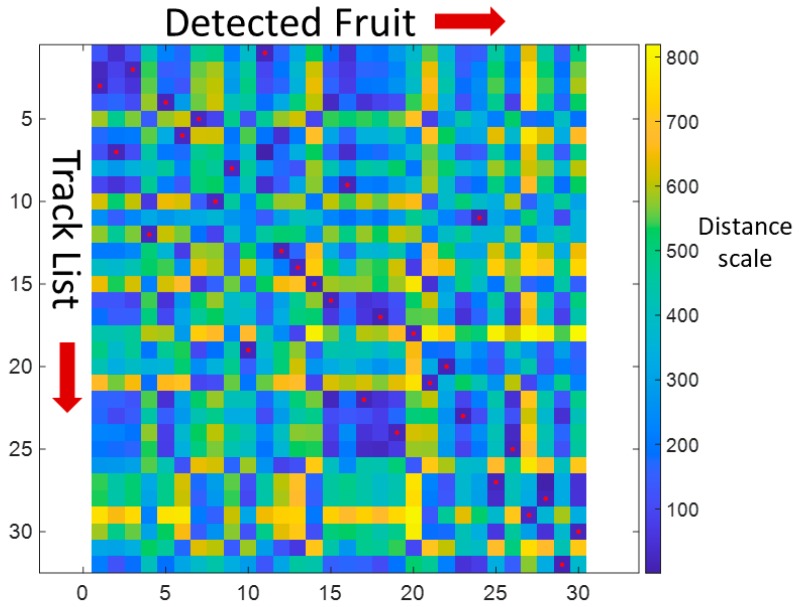
Hungarian assignment for a 32 × 30 cost matrix. X axis represents new fruit (jobs) detected in the image, while the Y axis represents previously detected fruit (workers) present in the track list. In this example data set, the minimum sum cost was 623.

In the example provided in Figure 3, there are 32 fruit in the track list and 30 fruit detected in the new image. The colour scale refers to the distance (in pixels) between the centroid of each new fruit and tracked fruit. The red dots signify the assignments of tracked fruit to new fruit, based on a minimum sum cost (Equation (11)). Tracked fruit # 26 and 31 failed to match a new fruit, so a trajectory will be borrowed from a neighbouring fruit to anticipate position in future frames, or these fruit will be removed from the track list if not detected in the past 15 frames (see Results for the rationale for choice of the value 15). As the individual cost for all tracked fruit was less than the threshold value (60, see Results), all assignments were considered to be valid.

**Figure 4:**
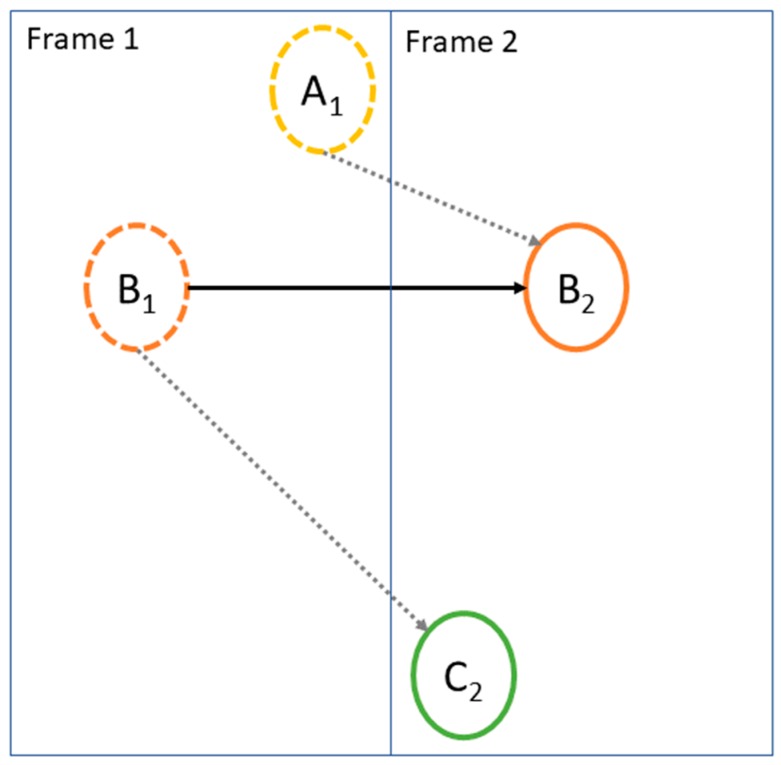
Example of multiple-to-one assignment for three fruit (A, B, C): both A_1_ and B_1_ are assigned to B_2_, but finally only the assignment of B_1_-to-B_2_ is valid as it has a smaller distance.

If the distance of a fruit in Frame 2 to a fruit in the previous frame was large (>60 pixels), the Frame 1 fruit will fail assignment. In the case shown in Figure 4, fruit A and B are detected in Frame 1 (as A_1_ and B_1_) and are tracked. In Frame 2, fruit A is not detected, fruit B is detected in position B_2_, and a new fruit C is detected at position C_2_. To obtain the global optimized (lowest) cost, the Hungarian algorithm will assign B_2_ to A_1_ and C_2_ to B_1_. This incorrect assignment results in an under-count of fruit, as noted in Table 1, column iv. Further, if the distance between B_1_ and C_2_ is greater than the distance threshold, the assignments is rejected. In this example, A_1_ was wrongly assigned to B_2_, the prediction models for fruit A and B wrongly updated, and a missed count result. 

To solve the issue described above, an improved Hungarian algorithm is proposed, in which:

(i) the Hungarian algorithm is applied to tracked and new fruit to obtain one-to-one assignments;
(ii) the maximum distance threshold is applied to decorrelate the assignments with large distances;
(iii) the Hungarian algorithm is applied a second time to unassigned tracked fruit and new fruit;
(iv) where two tracked fruit have been assigned the same new fruit (a ‘multiple-to-one assignment’), only the assignment with smaller cost (distance) is retained.

As in the case of Figure 4, the improved Hungarian algorithm can fail to count a new fruit in the situation that the new fruit appears very close to a tracked fruit that is not detected in the current frame, (i.e., smaller than 60 pixels). However, such cases were found to be rare.

The fruit correlation between frames can be lost due to an incorrect Hungarian assignment, temporary occlusion, or failure of detection. The Kalman filter was used to mitigate these issues by predicting the fruit location across subsequent frames. However, the Kalman prediction will lose accuracy in the prediction of fruit position as the frame number increases. Therefore, the maximum number of continuous unobserved frames that the fruit was carried on the track list was empirically set to 15.

**Figure 5:**
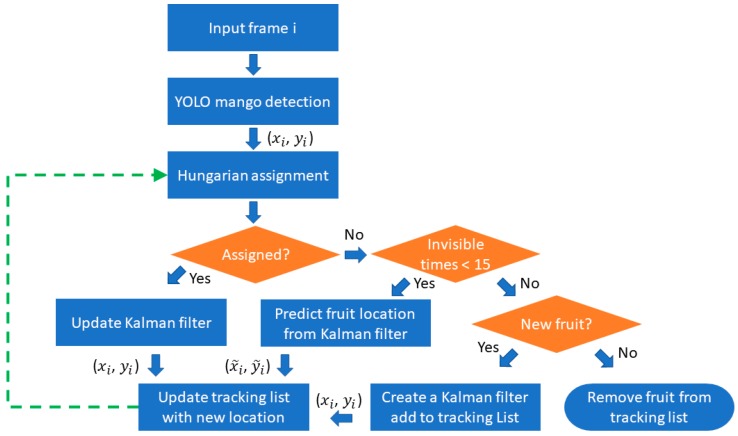
Workflow of the proposed method.

The workflow of the proposed method is illustrated in Figure 5. For an input video frame, MangoYOLO was used to detect fruit, then the adapted Hungarian algorithm was used to correlate to fruit detected in the previous frame. If a fruit was in the track list but not detected in the current frame, and if its continuous unobserved frames was less than a specified value ‘a’ (where a = 15, see Results), the location predicted by the improved Kalman filter was registered as the new location of the fruit. If the count was >a, the fruit was removed from the track list. If a fruit detected in the current frame was not in the track list, it was considered to be a new fruit, a new Kalman filter was created and the fruit was added to the track list. 

**Figure 6:**
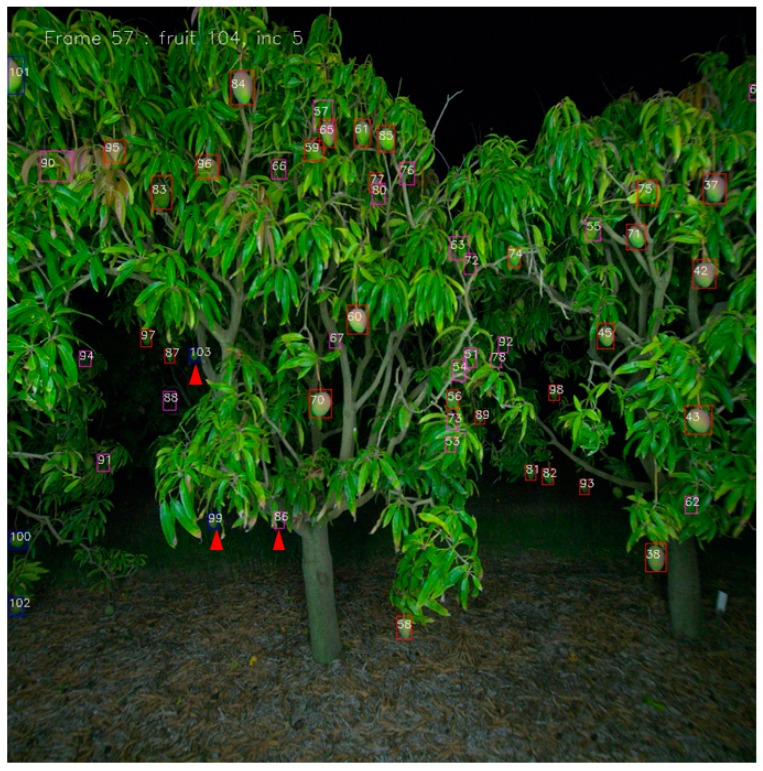
A tracking example: red bounding boxes indicate tracked fruit with valid assignments; pink indicates predicted location for fruit on the track list that were not detected in the current frame; blue indicates a new fruit detection. Numbering on fruit is in order of detection. Red arrows point to fruit mentioned in text.

A tracking example is illustrated in Figure 6. Each fruit was assigned a unique tracking number by its order of appearance. If a correlation (assignment) was found for a tracked fruit in the current frame, the fruit bounding box was coloured red. If there was no assignment for a tracked fruit in the current frame, the projected position of the missing fruit was shown with a pink bounding box. New fruit detections were shown in blue bounding boxes. Most new fruit occurred on the left side of the image, which is the direction of camera travel. However, fruit that were heavily occluded by foliage or other fruit could become visible from a specific angle, appeared in the centre or right of the image, i.e., fruit 103. If a fruit passed out of view for more than 15 frames or its predicted location become inaccurate relative to its actual location within 15 frames, it would be treated as a new fruit (i.e., double counted), as occurred for fruit 86/99. 

To reduce the number of fruit seen twice in imaging of the tree from both inter rows, a threshold was placed on fruit size, as fruit on the far side of the canopy in a given image appear smaller. Specifically, bounding boxes with width <12 pixels or height <15 pixels were excluded from detection. 

#### 2.2.3. Human Count

For reference values, fruit in the images were counted by a human, and the fruit number on trees was counted following harvest. For videos, the tracking results were examined frame by frame, and the count difference between the estimates of a target tree from its neighbouring trees were recorded as the fruit count of the tree. Errors in the association of fruit to individual trees will impact individual tree count but not total row count.

## 3. Results and Discussion

### 3.1. MangoYOLO Performance

**Figure 7:**
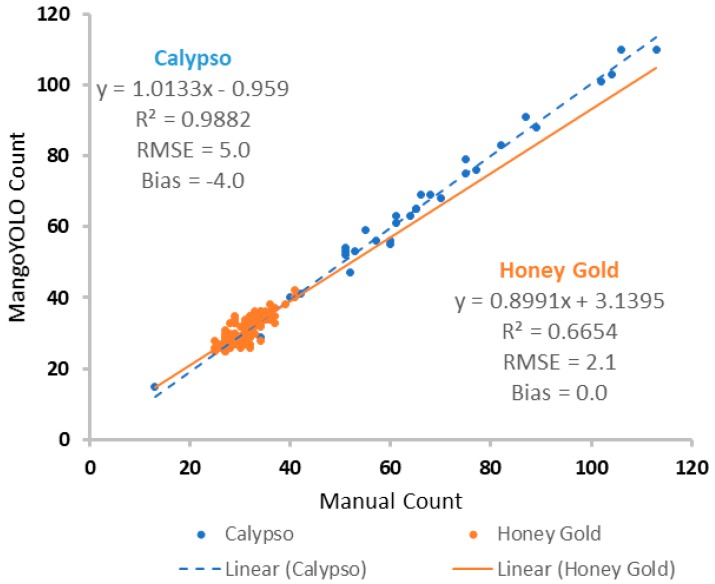
Scatter plot of MangoYOLO count against manual (human) count of fruit per image for images of Calypso^tm^ trees in 2018 (blue symbols), and video of HoneyGold^tm^ trees in 2018 (orange symbols).

The MangoYOLO model was trained on images from a mango orchard of the Calypso variety, collected in 2017 [32]. When tested on images from the same orchard during the subsequent season (2018), a R^2^ = 0.988, RMSE = 5.0, and bias = −4.0 fruit/image was achieved, relative to the human count (Figure 7). For the MangoYOLO model used in the assessment of the 110 video frames of cultivar HoneyGold, R^2^ = 0.665, RMSE = 2.1 and bias = 0.0 fruit/image (Figure 7). Thus the model performed well, irrespective of cultivar. The lower R^2^ for the video result was a result of the lower variation in fruit load number in these images, compared to that in the Calypso images. For the HoneyGold video, there was an average of 31 fruit detected per frame, with 1.4 false positives and 1.4 false negatives. The false positive errors of MangoYOLO resulted in an over-count while the false negative errors resulted in an under-count. The numbers of false positives and false negatives were similar, resulting in a low bias.

### 3.2. Choice of Maximum Unobserved Times and Threshold for Hungarian Assignment

**Figure 8:**
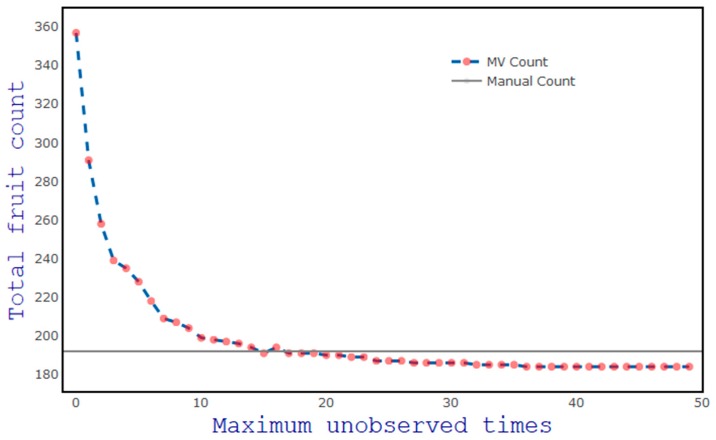
Impact of the value of maximum unobserved times on MangoYOLO fruit count in a video frame. The horizontal line denotes the human count of fruit in frame.

A range of values from 0 to 50 was considered for the number of frames a fruit was not detected in before the fruit was dropped from the tracked list (‘maximum unobserved times’), in the context of the impact on prediction accuracy (Figure 8). At a setting of 0, the Kalman filter was not used and only the Hungarian algorithm was used to correlate fruit. In this case, once a fruit loses its assignment in the subsequent frame, it was counted as a new fruit if detected in subsequent frames. This setting resulted in a high count (n = 357), compared to the human count of 192. Count numbers stabilised at a threshold of 15 unobserved frame times, with a MV count (n = 191) close to the human count (n = 192), and this value was adopted in subsequent work.

**Figure 9:**
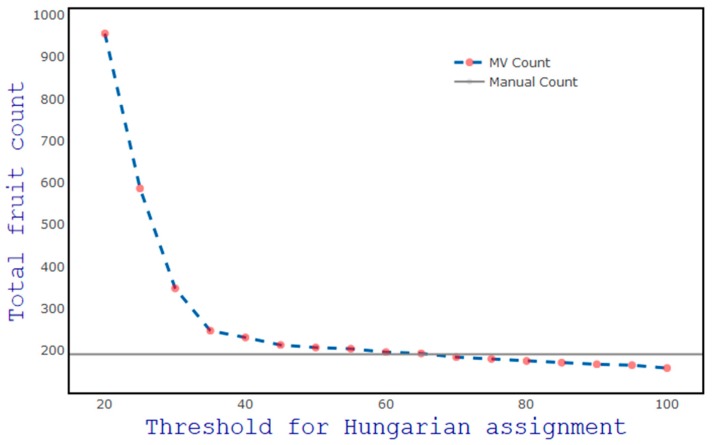
Impact of the value of threshold for Hungarian assignment on fruit count in the video. The horizontal line denotes the human count of fruit in frame.

The distance threshold used in assessing the validity of a Hungarian assignment was optimised by consideration of a range of values from 20 to 100 pixels, with a step of five (Figure 9). In principle, a smaller value should result in an increased repeat count, whereas a larger value should result in an increased incorrect assignment of new fruit to tracked fruit, leading to an underestimation of count. For example, if the platform speed was higher than the assumed 20 pixels per frame, at a setting of 20 pixels re-appearing fruit would be considered as new fruit, leading to over-counting (count of 956 fruit). The estimation was close to human count at a setting of 60 pixels (Figure 9).

### 3.3. Frame by Frame Comparison Between Human and Proposed Method

A frame-by-frame assessment of the fruit tracking and counting results of the MangoYOLO-Kalman filer-Hungarian algorithm method was undertaken by a human, for a video of 110 frames. Several kinds of errors were noted (Table 1)—(i) repeat count associated with temporary occlusion within a 15 frame interval, in which the predicted position is inaccurate; (ii) false prediction due to an inaccurate prediction model or the platform suffering an abrupt movement; or (iii) missing of a previous detection. These errors result in a fruit count overestimate. Another case can result in an underestimate—(iv) when a new fruit appears close to a tracked fruit and the tracked fruit does not exist in the current frame, the new fruit is assigned to the tracked fruit and a count is missed. Over the 110 frames assessed, the repeat fruit count was 9.7%, while the miss count was 7.1%, resulting in an over-estimate of 2.6% compared to the human assessment. Overall, the proposed method resulted in an estimate of “non-hidden” fruit that was 102.6% of the count of the harvest tally.

### 3.4. Fruit Count From Video

A comparison was undertaken of the harvest count to the dual-view image and MangoYOLO—Kalman filter—Hungarian algorithm method estimate, for a continuous row of 21 trees (Table 2). The dual view method achieved a count equivalent to 40% of the harvest count with bias corrected RMSE = 21.7 fruit/tree, while the proposed method achieved a count equivalent to 62% of the harvest count with bias corrected RMSE = 18.0 fruit/tree. 

### 3.5. Orchard Application

Any imaging system based on a ‘drive by’ imaging platform will suffer a failure to ‘see’ a proportion of fruit on the tree. As expected, the video based MangoYOLO—Kalman filter—Hungarian algorithm tracking method improved the detection of fruit per tree, by adding additional imaging perspectives. The ratio of total (harvest) count to machine vision count was decreased from 2.5 using the dual view method to 1.6 using the video based method. 

Thus the MV based estimation of mango fruit load, and ultimately automated harvest, is suited to orchards in which canopies are managed to have all fruit visible on the external wall of the canopy. This involves a move to canopies akin to the high density apple production systems, a trend that is already underway in the mango industry, e.g., [46]. The orchard imaged in the current study was a ‘difficult’ case, with a high proportion of ‘hidden’ fruit due a relatively thick, dense canopy, pruned to a continuous hedge. Future work should evaluate the technique in context of narrower canopy architectures, where hidden fruit becomes less of an issue and double counting of fruit from imaging from the two sides of the canopy becomes a greater issue. Another solution involves the generation of a correction factor for hidden fruit per tree, or indeed, per image based on characters within the image (e.g., foliage overlap).

## 4. Conclusions

The work of this study supports an automated imaging system for the estimation of mango orchard fruit load, extending a progression of studies [28,29]. The use of MangoYOLO [32] for mango detection and counting is validated in the current study. Video based Kalman tracking was demonstrated to provide an improved estimate of total fruit load over the dual view imaging procedure. Video based fruit yield estimation reduces the number of hidden fruit by providing more viewpoints of the canopy. 

Video based methods for fruit tracking and counting have been proposed by several contemporary research groups. These imaging systems are complex and costly, due to the use of GNSS/IMU or LiDAR to track camera movement or obtain precise real-word coordinates of individual fruit. Further, registration errors can impact the calculation of real-world coordinates and worsen the tracking of fruit. The practical adoption of an in-field machine vision solution to fruit load estimation requires a compromise between cost, ease of use, and accuracy/precision. 

The multi-view method presented in this study employed only LED lighting and a camera, and does not require differential GNSS if orchard rather than per tree estimates are required. The proposed method thus has lower hardware complexity and cost (e.g., the hardware cost of the current imaging rig of LED panel, camera and computer was <US$ 4000, while a LiDAR and dGNSS system can easily add >US$ 30,000). The system acquired 10 frames per second while that of Stein, et al. [30] employed 5 fps at a similar vehicle speed, providing more view-points per tree. The video based system does not allow for localisation within the orchard or for segmentation of trees, but could be used in the production of orchard maps given the use of GNSS or row locators, e.g., barcodes. The proposed method could be applied to other crops, such as apples and citrus.

## Figures and Tables

**Table 1:** Error attributions based on human assessment for fruit detection in 110 video frames using the tracking (MangoYOLO–Kalman filter–Hungarian algorithm) method.

*Human Count*	(i) Repeat Due to Occlusion in Previous Frames	(ii) Repeat Due to FN in Previous Frames	(iii) False Prediction of Position	Total Repeat Count	(iv) Missed Count Due to New Fruit Assigned to Old Fruit Position	*Estimated Count*
*192*	3	1	15	*19*	−14	*197*
*100%*	1.5%	0.5%	7.8%	*9.9%*	−7.3%	*102.6%*

**Table 2:** Fruit count of each of 21 trees based on the harvest fruit tally, dual view imaging count (as per [32]), and tracking (MangoYOLO–Kalman filter–Hungarian algorithm) method count. Correction factors refers to the ratio of MV count to harvest tally.

	Harvest	Dual-View Imaging	Tracking
Total (#fruit/21 trees)	3286	1322	2050
Average (#fruit/tree)	156.5	63.0	97.6
Bias (#fruit/tree)	-	−93.5	−58.9
% MV/harvest	-	40.2%	62.3%
RMSE-bc	-	21.7	18.0
Correction factor	-	2.5	1.6

## Supplementary Materials

The following are available online at https://www.mdpi.com/1424-8220/19/12/2742/s1, Video S1: Fruit Tracking Count.
